# ATP Binding Cassette Transporter Mediates Both Heme and Pesticide Detoxification in Tick Midgut Cells

**DOI:** 10.1371/journal.pone.0134779

**Published:** 2015-08-10

**Authors:** Flavio Alves Lara, Paula C. Pohl, Ana Caroline Gandara, Jessica da Silva Ferreira, Maria Clara Nascimento-Silva, Gervásio Henrique Bechara, Marcos H. F. Sorgine, Igor C. Almeida, Itabajara da Silva Vaz, Pedro L. Oliveira

**Affiliations:** 1 Laboratório de Microbiologia Celular, Instituto Oswaldo Cruz, Rio de Janeiro, Brazil; 2 Centro de Biotecnologia e Faculdade de Veterinária, Universidade Federal do Rio Grande do Sul, Porto Alegre, Brazil; 3 Programa de Biologia Molecular e Biotecnologia, Instituto de Bioquímica Médica Leopoldo de Meis, UFRJ, Rio de Janeiro, Brazil; 4 Departamento de Patologia Veterinária, Faculdade de Ciências Agrárias e Veterinárias, Universidade Estadual Paulista, Jaboticabal, Brazil; 5 Instituto Nacional de Ciência e Tecnologia em Entomologia Molecular–INCTEM, Rio de Janeiro, Brazil; 6 The Border Biomedical Research Center, University of Texas at El Paso, El Paso, Texas, United States of America; Kansas State University, UNITED STATES

## Abstract

In ticks, the digestion of blood occurs intracellularly and proteolytic digestion of hemoglobin takes place in a dedicated type of lysosome, the digest vesicle, followed by transfer of the heme moiety of hemoglobin to a specialized organelle that accumulates large heme aggregates, called hemosomes. In the present work, we studied the uptake of fluorescent metalloporphyrins, used as heme analogs, and amitraz, one of the most regularly used acaricides to control cattle tick infestations, by *Rhipicephalus (Boophilus) microplus* midgut cells. Both compounds were taken up by midgut cells *in vitro* and accumulated inside the hemosomes. Transport of both molecules was sensitive to cyclosporine A (CsA), a well-known inhibitor of ATP binding cassette (ABC) transporters. Rhodamine 123, a fluorescent probe that is also a recognized ABC substrate, was similarly directed to the hemosome in a CsA-sensitive manner. Using an antibody against conserved domain of PgP-1-type ABC transporter, we were able to immunolocalize PgP-1 in the digest vesicle membranes. Comparison between two *R*. *microplus* strains that were resistant and susceptible to amitraz revealed that the resistant strain detoxified both amitraz and Sn-Pp IX more efficiently than the susceptible strain, a process that was also sensitive to CsA. A transcript containing an ABC transporter signature exhibited 2.5-fold increased expression in the *amitraz-resistant* strain when compared with the susceptible strain. RNAi-induced down-regulation of this ABC transporter led to the accumulation of metalloporphyrin in the digestive vacuole, interrupting heme traffic to the hemosome. This evidence further confirms that this transcript codes for a heme transporter. This is the first report of heme transport in a blood-feeding organism. While the primary physiological function of the hemosome is to detoxify heme and attenuate its toxicity, we suggest that the use of this acaricide detoxification pathway by ticks may represent a new molecular mechanism of resistance to pesticides.

## Introduction

Ticks and tick-borne diseases began to be considered economic and public health concerns at the end of the nineteenth century, when the number of cattle increased in an attempt to feed a growing human population [1]. Tick-borne diseases were some of the first arthropod-borne diseases described. *R*. *microplus* is the major vector of *Babesia* spp. and *Anaplasma* sp., which cause severe economic losses in the largest cattle farms in tropical and subtropical countries [2]. In the midgut of ticks, hemoglobin from the blood of the vertebrate host is endocytosed by the so-called “digest cells,” and its degradation is accomplished by hydrolytic lisosomal-type enzymes inside acidic digestive vacuoles [3–6]. The free heme that is produced by this process is transferred from these vacuoles to the cytosol and subsequently moved to a very specialized organelle called hemosome, wherein heme aggregates accumulate [6]. This process is responsible for alleviating digest cells—and the tick as a whole—from the potentially deleterious effects of heme. In hematophagous insects, a wide array of adaptations have been reported that provide protection against heme toxicity and contribute to the adaptation of the animal to a diet based on vertebrate blood [7]. In the case of the digest cell of the tick midgut, however, an intracellular pathway dedicated to heme transport from digestive vesicles to hemosomes has been implicated as a key aspect of heme detoxification, although the molecular nature of the mechanisms involved in transport across cellular membranes or through the cytosol remains poorly understood.

Intracellular pathways involved in heme transport inside cells have been studied in several organisms. In some species of pathogenic bacteria, ABC transporters have been shown to transport heme obtained from the host [8, 9]. In the last few years, important advances have been made, including the discovery of three types heme transporters in eukaryotic cells: the feline leukemia virus receptor (FLVCR) [10, 11], intestinal heme transporter [12] and heme regulated genes 1–4 (HRG) [13]. In contrast, the participation of ABC transporters in heme movement across membranes in metazoan organisms is much less studied. In one occasion, evidence was obtained indicating that the BCRP/ABCG2 transporter functions as a heme exporter [14, 15]. A recent report provided for the first time conclusive experimental demonstration that an ABC transporter (an ABCC5) acts as a heme transporter in *C*. *elegans*, yeast, zebrafish and mammalian cells [16].

Drug detoxification in eukaryotic cells is generally described as a process that involves three steps: the chemical modification of xenobiotics, followed by conjugation to anionic groups such as glutathione, glucuronate or sulfate, and finally, excretion by ABC transporters [17]. Resistance to insecticides has been shown to involve enzymes from the first two classes, including esterases, P-450 cytochromes and glutathione-S-transferases [18–20], but the participation of ABC transporters in the development of resistance only recently has been acknowledged [21–23]. Here, we show that heme transport from the digestive vesicle to the hemosome is mediated by RmABCB10, an ABC transporter of the subfamily B10 that is upregulated in a tick cell line resistant to ivermectin [24] and is also up-regulated in an acaricide-resistant tick strain obtained from the field [25]. We also present evidence that a common acaricide, amitraz, is accumulated into the hemosomes through a pathway that uses the same ABC transporter, in this way outlining a novel type of resistance to pesticides.

## Materials and Methods

### Ethics Statement

All animal care and experimental protocols were conducted following the guidelines of the institutional care and use committee (Ethics Committee on Animal Experimentation of the Federal University of Rio Grande do Sul) and were approved under the registry # 14403/protocolo 07. Technicians dedicated to the animal facility at Faculdade de Veterinária from UFRGS carried out all aspects related to cattle husbandry under strict guidelines to insure careful and consistent handling of the animals.

### Animals

*R*.*microplus* of the Porto Alegre strain, (hereafter referred to as the POA strain), free of *Babesia* spp. and *Anaplasma* spp., were reared on calves obtained from a tick-free area and maintained at the Faculdade de Veterinária of Universidade Federal do Rio Grande do Sul (UFRGS), Brazil. Fully engorged adult females were kept in Petri dishes at 28°C and 80% relative humidity until use. The animals were handled in compliance with the UFRGS and EMBRAPA review committee for animal care. The *amitraz-resistant* strain of *R*. *microplus* (hereafter referred to as the Ibirapuã strain) was collected from a farm in the Ibirapuã district, Bahia state, Brazil, and maintained on calves reared in a tick-free area at Empresa Brasileira de Agropecuária (EMBRAPA, Gado de Leite) from Juiz de Fora (MG), Brazil. Amitraz resistance was evaluated using the adult immersion test (AIT), a bioassay applied to fully engorged female ticks, performed as described by Drummond *et al*. [26]. Briefly, groups of 10 females were immersed for 5 min in the acaricide solutions, usually using the acaricide concentrations recommended by the manufacturer. The control group was immersed in distilled water. The viability was estimated using the Porto Alegre strain as a susceptible standard, which showed 0% survival to all types of acaricides tested [27]. The effect of pesticides on the Ibirapuã strain, in contrast, ranged from a lack of resistance to some organophosphates, moderate resistance to pyrethroids and 100% survival to 85 μM of amitraz.

### Digest cell culture

The digest cell culture was performed as described by Lara et al. [6] Briefly, fully engorged females on the second day after a blood meal were rinsed in 70% (v/v) ethanol for 1 min and dissected in sterile phosphate buffered saline (PBS) containing 200 U mL^–1^ streptomycin and penicillin. Midguts were isolated in sterile Petri dishes, and digest cells were detached from the gut wall with sterile tweezers. The cells were carefully collected using a Pasteur pipette, washed three times in the same buffer and then placed in a 12-well culture plate with L-15 Leibowitz's medium supplemented with 150 mM NaCl plus streptomycin and penicillin, both at 100 U mL^–1^. The cells were kept at 28°C in the dark until use.

### Uptake of metalloporphyrins and Rhodamine 123 by digest cells

In a previous report [6] we used a fluorescent metalloporphyrin, palladium mesoporphyrin, as a fluorescent heme analog to characterize heme intracellular pathways in the digest cells of *R*. *microplus*. Here, we used two other metalloporphyrins as heme analogs, tin-protoporphyrin IX (Sn-Pp IX) and zinc-protoporphyrin (Zn-Pp IX), as the fluorescence of these compounds exhibits a higher quantum yield than the palladium complex (data not shown). A 20 mM stock solution of Sn-Pp IX was prepared in DMSO and further diluted 1:1 with 0.1 N NaOH immediately before its addition to cells. Solutions (100 μM) were prepared by diluting the stock solution directly into culture medium. Fluorescence spectra were collected using an Eclipse 100 spectrofluorimeter (Varian, Palo Alto, USA) and showed two excitation peaks at 410 nm and 550 nm. The emission spectrum of both porphyrins showed a strong red fluorescence, with peaks at 582 nm and 630 nm. Zn-Pp IX was used in the artificial feeding of partially engorged ticks for RNA interference experiments, as described below.

The fluorescent images of Sn-Pp IX and Zn-Pp IX uptake by digest cells were obtained using a 100 W mercury lamp as the excitation light source with a Zeiss-15 filter set (BP 546–12 nm/FT 580 nm/LP 590 nm) and an Axioplan 2 microscope (Zeiss, Gottingen, Germany). In experiments to observe Sn-Pp IX uptake, the cells were preincubated or not in culture medium containing 10 μM of the ABC inhibitor indicated. After 2 h, 100 μM of Sn-Pp IX was added to the medium. Images were acquired after 4 h of incubation. To study the uptake of Rhodamine 123, a canonical ABC transporter superfamily substrate, cells were incubated with 0.5 μM of Rhodamine for 4 h Images were acquired using an Olympus IX81 microscope with a Disk Spinning Unit type 3 (DSU) with a CellR MT20E Imaging Station equipped with a IX2-UCB controller and an ORCAR2 C10600 CCD camera (Hammamatsu). Image processing was performed with the Xcellence RT version 1.2 Software. Optical slices of 0.2 μM were generated with the DSU using a #52019 filter set (exc. 565/25x nm em. 620/25x nm, 51019BS). Quantitative analysis was made by blindly choosing circular portions of image with an area of 100 μm2 area, inside the digest cell in the bright field images and fluorescence was evaluated using ImageJ software.

### HPLC analysis of the accumulation of Sn-Pp IX and amitraz in the hemosome

Attempts to measure metalloporphyrin and amitraz uptake using primary digest cell culture failed because we did not manage to develop a reliable protocol to normalize the amount of cells in the culture, due to the variability in the amount of cell debris found in the medium. As mentioned above, heme accounts by at least 90% of dry weight of the isolated hemosomes [28], the final destination of the heme and amitraz trafficking pathway being studied here. Therefore, we choose to normalize incorporation of the fluorescent label or the acaricide relative to the mass of heme found in a hemosome preparation, which is approximately equivalent to normalize it relative to the mass of the isolated organelle. Digest cells from resistant or sensitive strains were placed in culture media and pre-treated for 30 min with ABC transporter superfamily modulators such as 10 μM CsA, 300 μM indomethacin, 50 μM verapamil, or 50 μM of trifluorperazine. After this preincubation, 50 μM of Sn-Pp IX or 35 μM of amitraz was added to the media to be taken up by cells. A control was performed by incubating the cells at 4°C to decrease metabolic activity. Hemosomes were isolated using a modification of the protocol described by Lara et al [28]: briefly, after 30 min of incubation with amitraz or Sn-Pp IX, the cells were disrupted by repeatedly pipetting with a 100 μL automatic pipette. Hemosomes were purified through centrifugation at 100 × *g* for 2 min in a 30% (w/v) sucrose cushion. The pellets formed below the cushions were dissolved by the addition of 0.025% (v/v) ammonium hydroxide and 5% (v/v) acetonitrile and vortexing. The suspensions were centrifuged at 14000 × *g* to remove insoluble particles, and the supernatant was applied to a Poros reverse-phase HPLC column (C8-C18; Applied Biosystems, California, USA) using 0.025% (v/v) hydroxide ammonium and a gradient of 5 to 100% (v/v) acetonitrile as the mobile phase. HPLC was performed using a diode array detector (SPD-M10A, Shimadzu, Tokyo, Japan) with an HPLC system LC-10AT (Shimadzu, Tokyo, Japan). Spectra from the heme, Sn-Pp IX and amitraz peaks were recorded during chromatography. The relative amount of Sn-Pp IX and amitraz found in hemosomes from digest cells incubated with these compounds was calculated using the heme content of hemosomes as an internal reference. To normalize the data, the amount of Sn-Pp IX or amitraz found in digest cells from the acaricide-resistant strain after 2 h exposure was defined as 100%. Absorbance values at 400 nm for heme and Sn-Pp IX and at 290 nm for amitraz had accumulated inside and the area under the corresponding peaks were used to calculate the relative amount of amitraz and Sn-Pp IX that were accumulated inside the hemosomes.

### Immunolocalization

Immediately after removing ticks from the bovine host, tegument of each parasite was carefully perforated with fine needles and was then immersed in buffered formalin, pH 7.0 for 12 h. After fixation, ticks were dehydrated for 30 min in each of the following ethanol concentrations: 70%, 80%, 90% and 100% and then were embedded in paraffin. Slides were deparaffinized in xylene and hydrated through graded alcohols. Antigen retrieval was achieved by steam heating in Tris–EDTA buffer plus 0.05% (v/v) Tween-20, pH 9.0, for 30 min. Tissues were blocked with 5% (w/v) BSA and 0.01% (w/v) Triton X-100 in PBS for 1 h, and then incubated for 12 h at 4°C with polyclonal rabbit antibody against a peptide corresponding to the first 50 aminoacid residues of the human P Glycoprotein (ABCB1) transporter (Abcam 129450); diluted 1:50 in PBS with 5% (w/v) BSA. The use of this heterologous antibody produced in a western blot assay bands similar to those expected according to the manufacturer datasheet (not shown). The sections were then washed and stained with Alexa-633 conjugated anti-rabbit secondary antibody (Sigma, Missouri, USA) diluted 1:200 in PBS containing 5% (w/v) BSA for 4 h and then observed in an Olympus IX81 microscope with a Disk Spinning Unit type 3 (DSU) with a CellR MT20E Imaging Station equipped with a IX2-UCB controller and an ORCAR2 C10600 CCD camera (Hammamatsu). Image processing was performed with the Xcellence RTversion 1.2 Software. Optical slices of 0.3 μm were generated with the DSU using a #52019 filter set (exc. 565/25x nm em. 620/25x nm, 51019BS).

### Histochemical staining of ATPase activity

Midgut sections of engorged females dissected on the third day after completion of the blood meal (ABM) were fixed in 1% (w/v) glutaraldehyde and 0.0005% (v/v) Triton X-100, 50 mM HEPES, pH 7.2, for 10 min at 4°C. Subsequently, the tissues were incubated with a reaction mixture composed of 5 mM ATP, 2.5 mM MgCl_2_, 5 mM CeCl_3_, 50 mM KCl, and 50 mM HEPES, pH 7.2, for 2 hours at 28°C, as described by Hulstaert et al. (1983). The samples were further fixed in 2.5% (w/v) glutaraldehyde, 4% (w/v) paraformaldehyde, and 50 mM HEPES, pH 7.2, for 72 hours at 4°C. After gradual dehydration in acetone, the tissues were embedded in Epon resin. Ultra-thin sections (60 nm) were observed under a Morgagni 268 transmission electron microscope (Fei, Oregon, USA) operating at 80 Kv.

### RNA extraction and cDNA synthesis

Midgut or digest cells were homogenized in TRIzol reagent (Invitrogen). After extraction following the manufacturer’s recommendations, RNA was treated with DNase I (Invitrogen, USA), and the concentration and quality of the RNA was estimated using a NanoDrop 1000 (Thermo Fisher Scientific, USA). For cloning experiments, the total RNA was reverse-transcribed using an oligo-dT primer and SuperScript II (Invitrogen, USA), and for quantitative PCR, total RNA was reverse-transcribed using the High-capacity cDNA Reverse Transcription kit with random primers, according to the manufacturer’s recommendations (Applied Biosystems, USA). cDNA was stored at -80°C until use.

### Cloning and sequence analysis of RmABCB10 transcript

To obtain the full-length sequence of RmABCB10, 5’RACE was performed using the 5’RACE System for Rapid Amplification of cDNA Ends kit (Invitrogen) according to the manufacturer’s directions. Total RNA prepared from gut digest cells was reverse transcribed using a gene-specific primer (ABCBm-GSP1- CGAATTTATAGAAGACCTTC) designed from the partial RmABCB10 sequence available (Genbank accession number JN098446.1). cDNA was purified, a homopolymeric tail (dCTP) was added to the 3' end of the cDNA using a terminal deoxynucleotidyl transferase and PCR was performed with a nested gene-specific primer (ABCBm-GSP2- GAGAACATTCTTTATGGTGCAAAGAATATGGAGGAAAGCTC) and the Abridged Anchor Primer (Invitrogen). cDNA amplification products were cloned into the pGEM-T vector (Promega), and the positive clones were sequenced using vector-specific primers (T7 and SP6) and two internal primers: 5’-AGCTTCTGACTGGACAGTAG-3’ (sense) and 5’-CCCACCGTAATAGAGGAC-3’ (antisense). The full-length sequence was deposited in NCBI with the accession number JN098446.2.

The predicted amino acid sequence of full-length RmABCB10 was searched for the presence of the ABC signature, and the Walker A and Walker B conserved motifs using the Conserved Domain search program available on the NCBI website. Multiple sequence alignments and phylogenetic analyses were performed using the MUSCLE algorithm [29] using the default settings in MEGA software version 5 [30]. SwissModel First Approach Mode was used for tertiary structure modeling. Membrane topology was predicted using SOSUI and TMHMM [31–33]. The sequences used in sequence alignments and phylogenetic analyses were *R*. *microplus* RmABCB10 (AEI91123.2), RmABCC1 (AEI91124.1) and RmABCC2 (AEI91125.1); *D*. *melanogaster* CG3156 (NP569844.2), MDR65 (NP476831.1), CG2613 (AAF59366.1), CG1703 (NP572736.1), CG1718 (NP608445.1) and CG2759 (NP476787.1); *D*. *pulex* 347264 (EFX85237.1), 347281 (EPX72783.1), 347357 (EFX73813.1), 347330 (EFX8324.11), 189585 (EFX66734.1), 312055 (EPX87570.1), and 347276 (EFX65703.1); *H*. *sapiens* ABCE1 (NP001035809.1), ABCG1 (NP058198.2), ABCB10 (NP036221.2), ABCB8 (NP009119.2) and ABCB7 (NP004290.2); *M*. *musculus* ABC-me(NP062425.1), and *S*. *cerevisiae* Mdl1p (NP13289.1), Mdl2p (NP015053.2) and Atm1p (NP014030.1).

### Real-time PCR

For quantitative analysis of mRNA expression levels using real-time PCR, two specific primers were designed to amplify a 95 bp fragment (5’- GCCGCAGTTGTCACTTGTTGGTTTG-3’ and 5’-ACGTCCGCTGCCACTTGCCTC-3’) of RmABCB10. A fragment of the β-actin gene that produces a 205 bp amplicon was used as a reference (5’GAGGAAGTACTCCGTCTGGATCGGCG3’ and 5’CCGTAGGGTGGCGTTGCCGG3’) [34]. Cycling parameters were 10 min at 95°C followed by 40 cycles of denaturation at 95°C for 15 s, annealing at 60°C for 15 s and extension at 72°C for 20 s. After amplification was complete, a melting curve analysis was performed using the default parameters of the instrument. Primer efficiency was measured with 6-fold serially diluted cDNA in triplicate. All samples were analyzed in triplicate. The relative expression ratio of RmABCB10 gene in each experiment was calculated according to the mathematical model described by Pfaffl [35] and used in the Relative Expression Software Tool (REST-MCS, version 2) [36].

### RNA interference

The full-length RmABCB10 sequence (1964bp) was amplified from gut cells cDNA using the primers 5’-ATGAACCCTACAGTTGAGTCCCAC-3’ (sense) and 5’-TCACCCAGACATTCTCTCATCATGTAAC-3’ (antisense) and cloned into the pGEM-T vector (Promega). A 578-bp fragment of RmABCB10 was amplified by PCR from the recombinant plasmid with gene specific primers containing the T7 promoter recognition sites (sense: 5’-GGATCCTAATACGACTCACTATAGGGGCTCTCAGTTGTTTCGTC-3’) (antisense: 5’- GGATCCTAATACGACTCACTATAGGGACATCCCAACCAGCG-3’). The PCR product was purified using a Gene Jet PCR Purification Kit (Fermentas) and used to synthesize dsRNA with a T7 Ribo Max Express RNA Kit (Promega) according to the manufacturer’s protocol. The dsRNA synthesis was evaluated by 1.5% (w/v) agarose gel electrophoresis, and the concentration was determined spectrophotometrically at 260 nm in a NanoDrop 1000 instrument (Thermo Fisher Scientific, USA).

The dsRNA of a tick-unrelated gene, MSP1 from *Plasmodium falciparum* (accession number AF061132), was used as a negative control (dsCont). This gene was obtained from a plasmid kindly provided by Dr. Gerhard Wunderlich (Instituto de Ciências Biomédicas, USP, Brazil). The 1110 bp sequence was recovered after digestion of the plasmids with Fast Digest SpEI (Fermentas), and the dsRNA of MSP1 was synthesized as described above. Partially engorged tick females (weighing between 25 and 70 mg) of the POA strain were manually removed from experimentally infested bovines. Groups of 15 tick females were immobilized on a glass plate covered with double-sided adhesive tape, and dsRNA solutions were injected into the hemocoel (1 μL, 5 μg/tick). These ticks were artificially fed using microhematocrit capillary tubes filled with blood from non-infested bovines collected in the presence of sodium citrate [25, 37]. Initially, females were fed with 50 μL of blood supplemented with either the fluorescent heme analog ZnPP diluted in DMSO (1:200, 100 μM) or DMSO alone (1:200), as a control for autofluorescence. After this initial meal, the females were fed until repletion with blood alone, without metalloporphyrin or DMSO. The females were allowed to feed for approximately 28 h, and then kept in separate vials at 27–28°C and 80–90% relative humidity. After 24 h or 48 h after feeding, the females were dissected, and the midguts were collected for RNA extraction and microscopy.

### Statistical analysis

Multiple comparisons were performed by a one-way ANOVA analysis of variance and an *a posteriori* Tukey’s test for pair-wise comparisons. Single comparisons were performed by T test analysis with Mann-Whitney test, using GraphPad Prism version 4.0 for Windows (GraphPad Software, San Diego, CA, USA).

## Results

**Fig 1:**
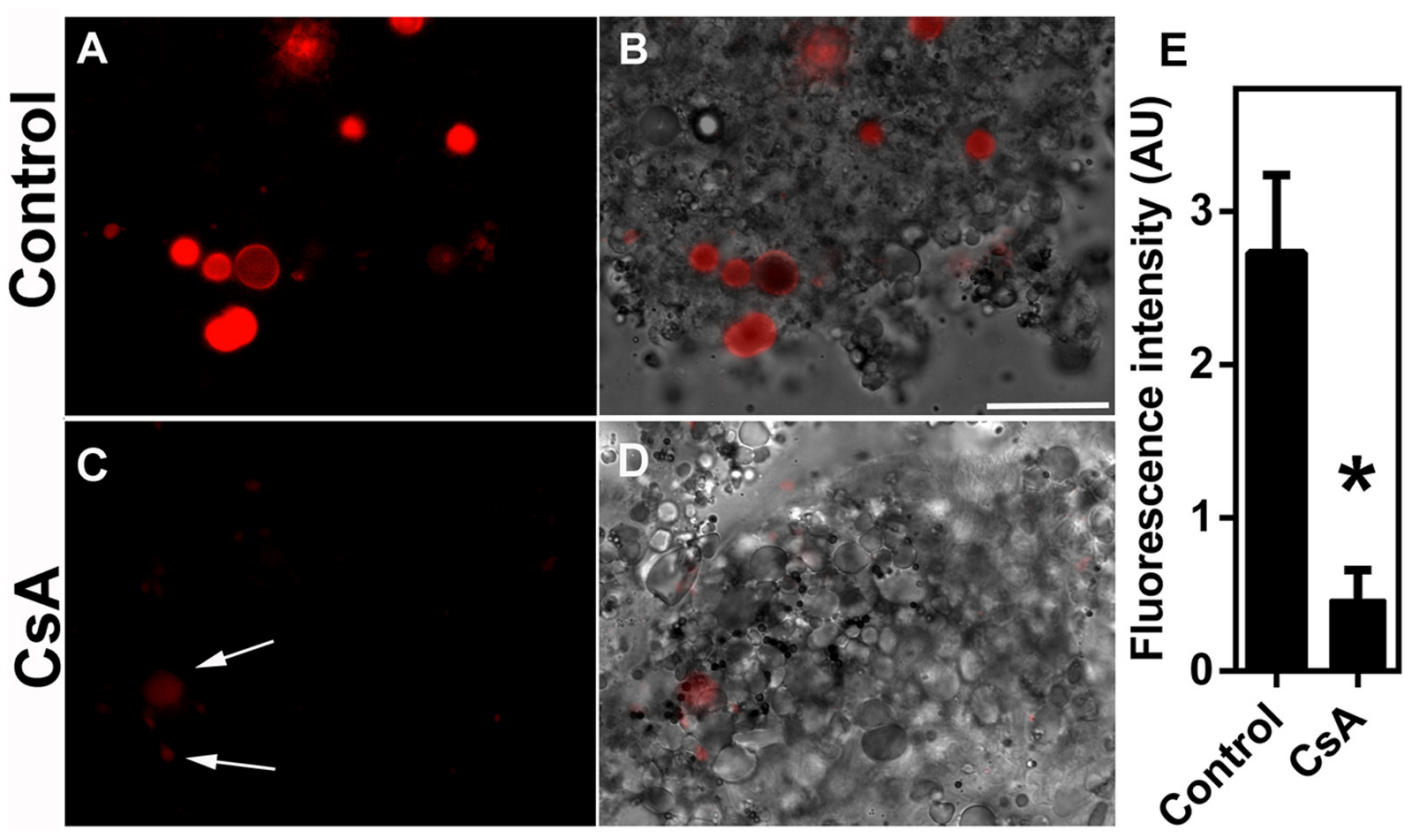
Uptake of Rhodamine 123 by midgut digest cells. Digest cells from fully engorged adult females were obtained as described in the Methods. Digest cells were incubated in the presence of 0.5 μM Rhodamine 123 for 4 h. (A-B) Control; (C-D) cells preincubated with 10 μM CsA. Panels are fluorescence images (A and C) or differential interference contrast (DIC) merged with fluorescence (B and D). Digestive vesicles labeled are indicated in the figure by white arrows. Scale bar is 40 μm. Fluorescence intensity was measured in images from two independent experiments (E). Data shown are mean ± SEM (n = 12). * means p < 0.05 (Student’s t test).

**Fig 2:**
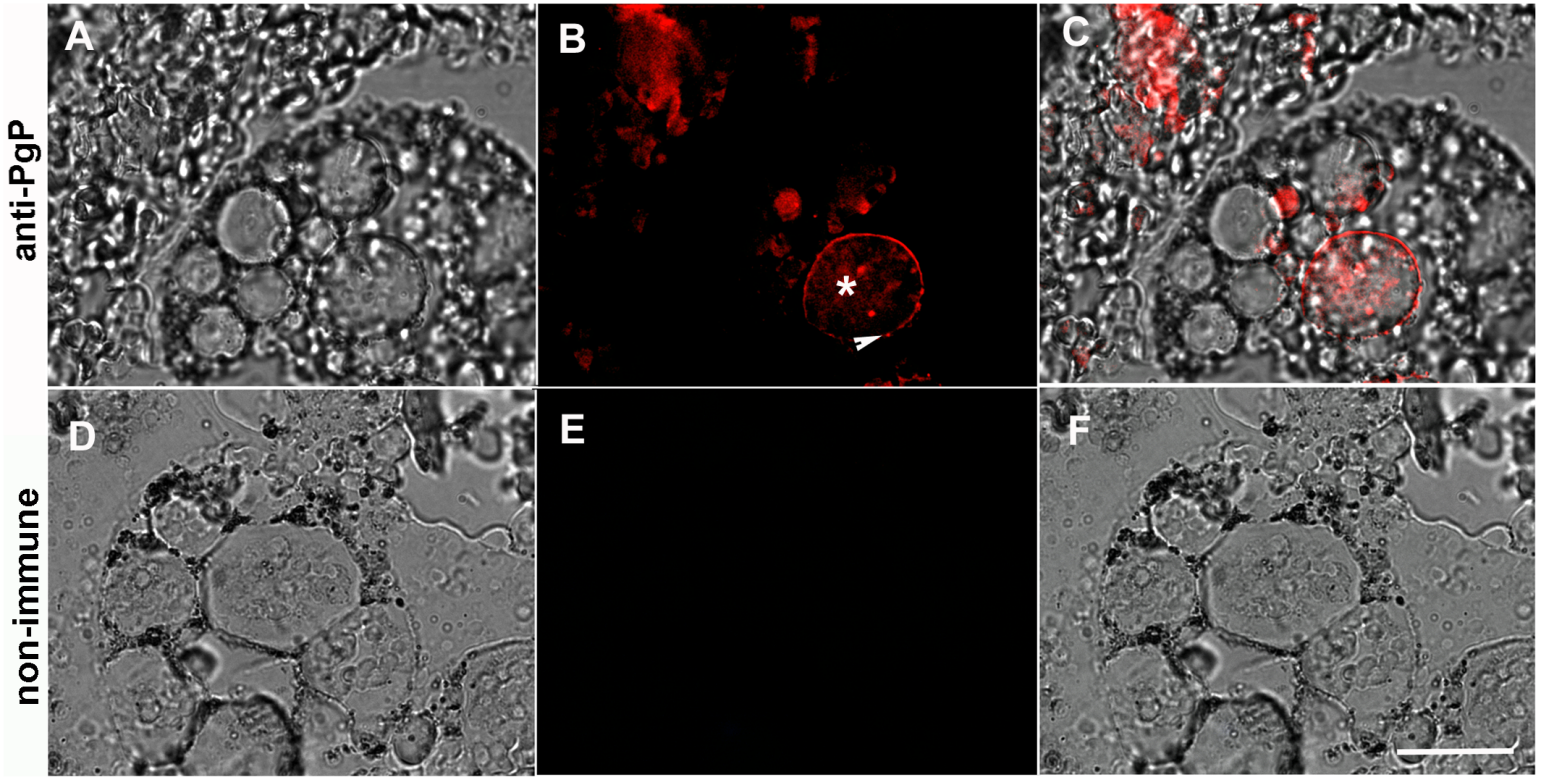
Immunolocalization of ABC transporters in the tick midgut digest cell. Fully engorged females were dissected, fixed and included in paraffin. Deparaffinized sections were stained using polyclonal antibodies against an aminoterminal segment of human PgP-1 (A-C) or rabbit non-immunized serum (D-F), followed by Alexa 633-labeled secondary antibodies. Images are DIC (A and D), fluorescence (B and E) and DIC merged with fluorescence (C and F). Asterisk shows a labeled digestive vesicle. Arrow indicates a labeled digestive vesicle membrane. The scale bars is 40 μm.

**Fig 3:**
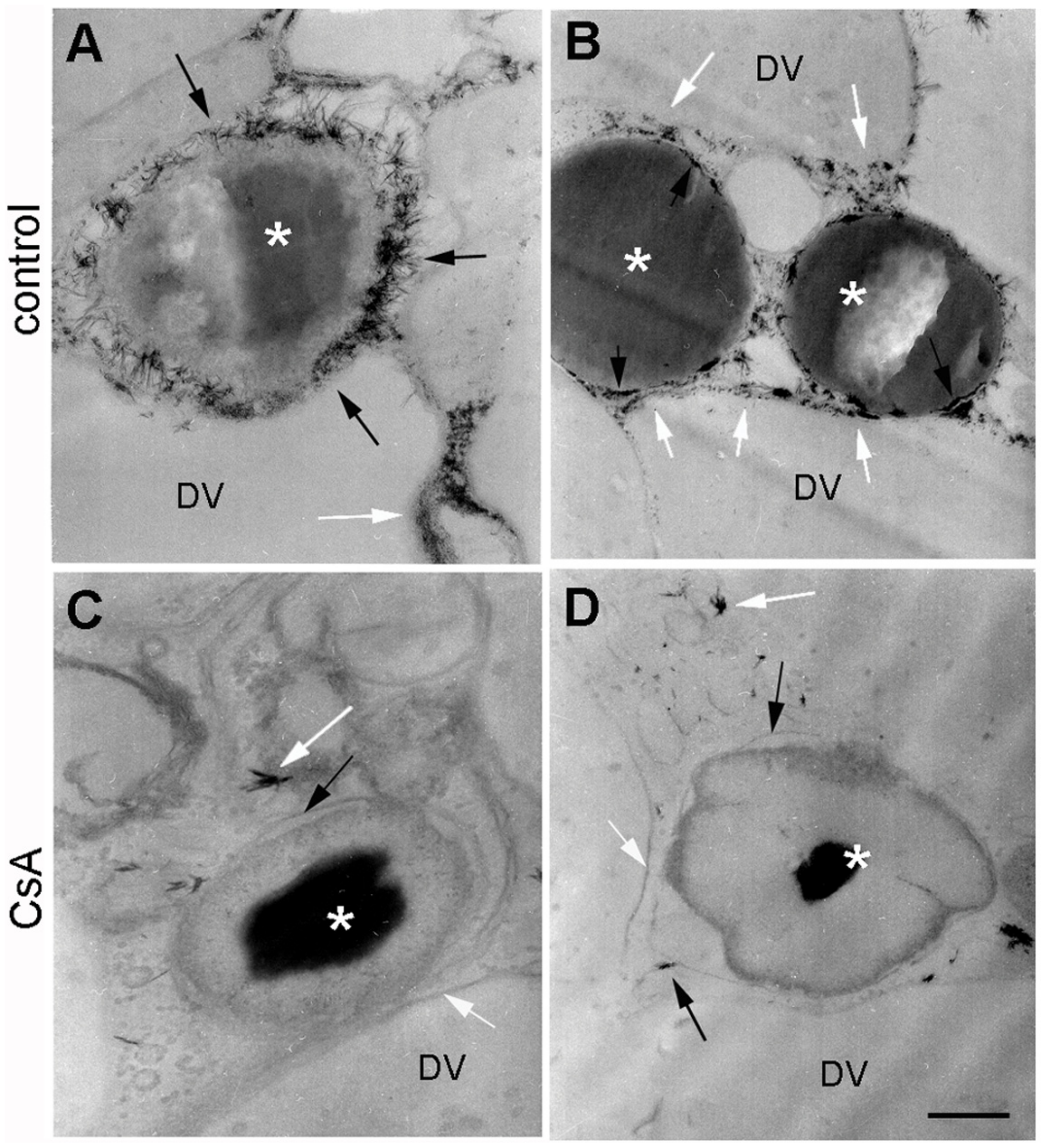
Identification of a CsA-sensitive ATPase activity in the hemosome membrane. (A-B) Digest cells from fully engorged tick females 2 days after blood meal showing hemosome (asterisk) and digestive vesicle (DV). Both hemosomes membranes (black arrows) and digestive vesicles membranes (white arrows) exhibit strong ATPase activity, as revealed by the precipitation of cerium phosphate (that appear as electron-dense precipitates near both membranes); (C-D) section from a distinct midgut diverticulum from the same tick, preincubated with 10 μM CsA for 30 min before the ATPase assay. The scale bar is 200 nm (B) or 100 nm (A, C and D). The images are representative of two independent experiments.

Digest cells of the tick midgut degrade hemoglobin and direct heme into an intracellular pathway that involves digestive vacuoles and ultimately leads to heme accumulation in hemosomes. To test whether this heme intracellular transport is dependent on an ABC transporter, digest cells isolated from the midgut of *R*. *microplus* were incubated with Rhodamine 123, a PgP protein (ABCB) transporter substrate that has been validated for membrane transport studies [38, 39]. The fluorescent dye was found to be associated with both digestive vacuoles (asterisks) and hemosomes (White arrows inset) after incubation (Fig 1A and 1B). Furthermore, uptake was markedly inhibited by preincubation with 10μM CsA, a commonly used ABC inhibitor [40, 41], which suggests the participation of a transporter of this type (Fig 1). This conclusion was also supported using immunocytochemistry with a commercially available polyclonal antibody against vertebrate PgP-1 ABC-transporter. The anti-PgP-1 antibody specifically labeled digest cell membranes (Fig 2A–2C). Further demonstration of the presence of an ABC ATPase activity in the digestive vesicle membrane was obtained by a cerium-based histochemical method [42]. In this method, phosphate released by ATPase activity complex with cerium, a heavy metal atom whose phosphate salt is highly insoluble [43]. Digestive vesicles (DV) exhibited high ATPase activity associated with their membranes (Fig 3A and 3B, black and white arrows, respectively) which is inhibited by CsA (Fig 3C and 3D). Some residual activity, however, was still found even in the presence of CsA (Fig 3C and 3D). Hemosomes presenting different electron densities in the heme aggregate found in the central area of the organelle (Fig 3, asterisks), probably reflect the gradual increase in heme content that occurs during maturation of the organelle, as proposed by Lara et al [28].

**Fig 4:**
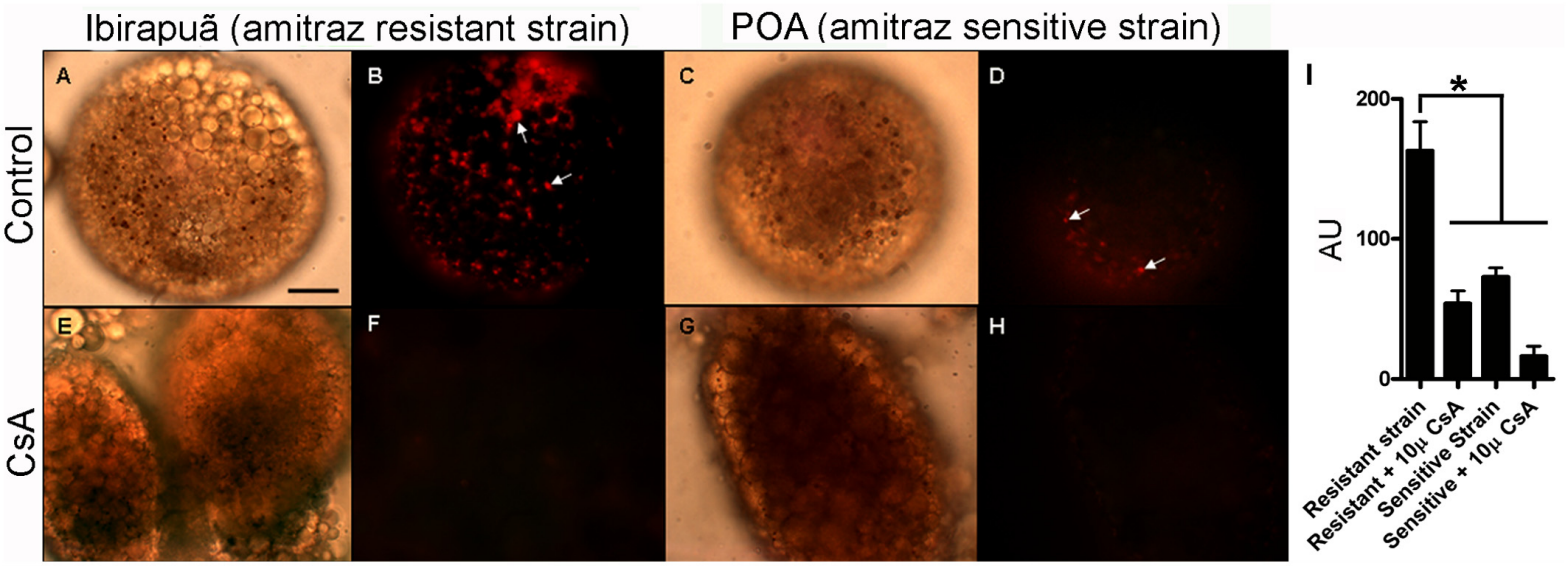
CsA-sensitive uptake of Sn-Protoporphyrin IX (Sn-Pp IX) is higher in amitraz-resistant ticks. Digest cells from tick strains sensitive or resistant to amitraz were incubated in the presence of 100 μM Sn-Pp IX for 2 h. (A,B) Amitraz-resistant strain; (C,D) amitraz-sensitive strain; (E,F) amitraz-resistant strain preincubated with 10 μM CsA; (G,H) amitraz-sensitive strain preincubated with 10 μM CsA. A, C, E and G are DIC images. B, D, F and H are fluorescence images of the metalloporphyrin. Arrows indicate Sn-Pp IX fluorescence associated with hemosomes. The scale bar is 60 μm. (I) Quantitative analysis of Sn-Pp IX uptake measured as fluorescence intensity of digest cells from resistant and sensitive strains (expressed in arbitrary units; AU). Data shown are mean ± SEM from groups of 10 randomly chosen images obtained from three independent experiments; * means p < 0.001 (one-way ANOVA followed by Tukey’s test).

**Fig 5:**
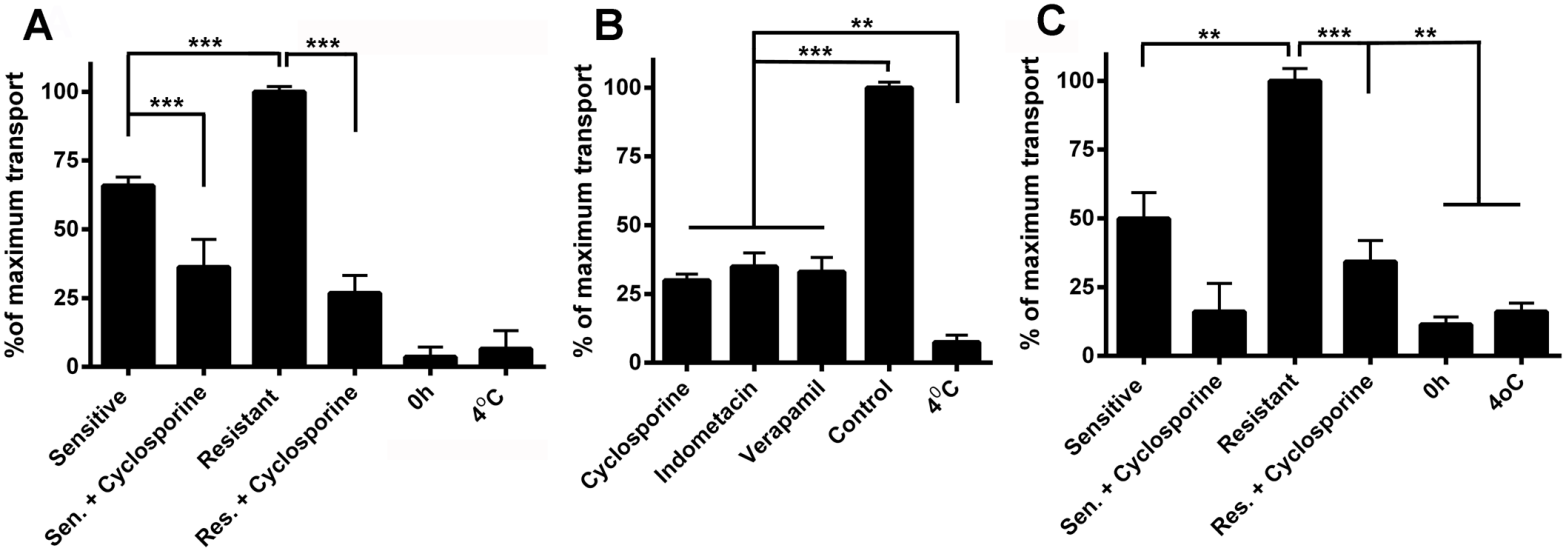
Sn-Protoporphyrin IX and amitraz accumulation into hemosomes is due to ABC transporters. Digest cells were incubated either with 10 μM Sn-Pp IX (A, B) or 10 μM amitraz (C), and hemosomes were isolated as described in the Methods. Hemosomes were fractionated by reverse phase HPLC chromatography, and the Sn-Pp IX or amitraz content was calculated in relation to the area of the heme peak, with the highest value set at 100%. (A) Cells were collected from either the resistant strain or the sensitive strain and were preincubated or not with 10 μM CsA and the Sn-Pp IX content was evaluated. (B) Cells were collected from the amitraz-resistant strain, and all inhibitors were used at 10 μM. The Sn-Pp IX content was evaluated. (C) Cells were collected from either the resistant strain or the sensitive strain and were preincubated or not with 10 μM CsA, and the amitraz content was evaluated. Incubations of the cells from the resistant strain at 4°C were included in all panels as controls lacking metabolic activity. * means p < 0.001; **means p < 0.05; *** means p < 0.01 (one-way ANOVA followed by Tukey’s test). Data are the mean ± SEM (n = 3).

In a previous report, it was determined that an ABC transporter was involved in the detoxification of ivermectin and other acaricides [24, 25, 44]. We therefore hypothesized that the presence of ABC transporters in the heme transport pathway that ends in the hemosome could also turn this organelle into a site for the disposal of other toxic compounds such as xenobiotics, in addition to its primary role as a site for the disposal of excess heme. As we had one *R microplus* isolate that was amitraz-sensitive, the POA strain and also an amitraz-resistant field isolate (the Ibirapuã strain), we compared the uptake of Sn-Pp IX by digest cells from both strains to observe the relationship between amitraz resistance and heme detoxification by ABC transporters. After 2 h of incubation in medium with Sn-Pp IX, digest cells of the amitraz-resistant strain presented higher metalloporphyrin uptake than cells from the susceptible strain (Fig 4B, 4D and 4I, white arrows). Sn-Pp IX uptake was markedly inhibited by CsA, which reinforces the conclusion that ABC transporters are involved in this process (Fig 4F, 4H and 4I). When cells were incubated with SnPp IX and the amount of SnPp IX in isolated hemosomes was evaluated by HPLC, organelles from the resistant strain also exhibited a higher level of accumulation (Fig 5A).

Cyclosporine A (CsA), verapamil and indomethacin are known to interfere with the function of ABC transporters CsA and verapamil are inhibitors of PgP proteins (ABCB transporters) and indomethacin is an inhibitor of MRP proteins (ABCC transporters) [45, 46]. All three inhibitors were able to reduce the relative amount of SnPp IX that reached the hemosomes (Fig 5B), confirming that members of this class of transporter are involved in the transport of metalloporphyrin into the hemosome. When added to the cell culture medium, amitraz was also sequestered into hemosomes, and uptake of this pesticide was higher in the amitraz-resistant Ibirapuã strain (Fig 5C). In both strains, SnPp IX transport was sensitive to CsA (Fig 5C). In the experiments shown in Fig 5, low temperature incubations (4°C) were performed as a control to confirm that uptake was dependent on active metabolism, and low levels of accumulation of SnPp IX were observed under these conditions.

**Fig 6:**
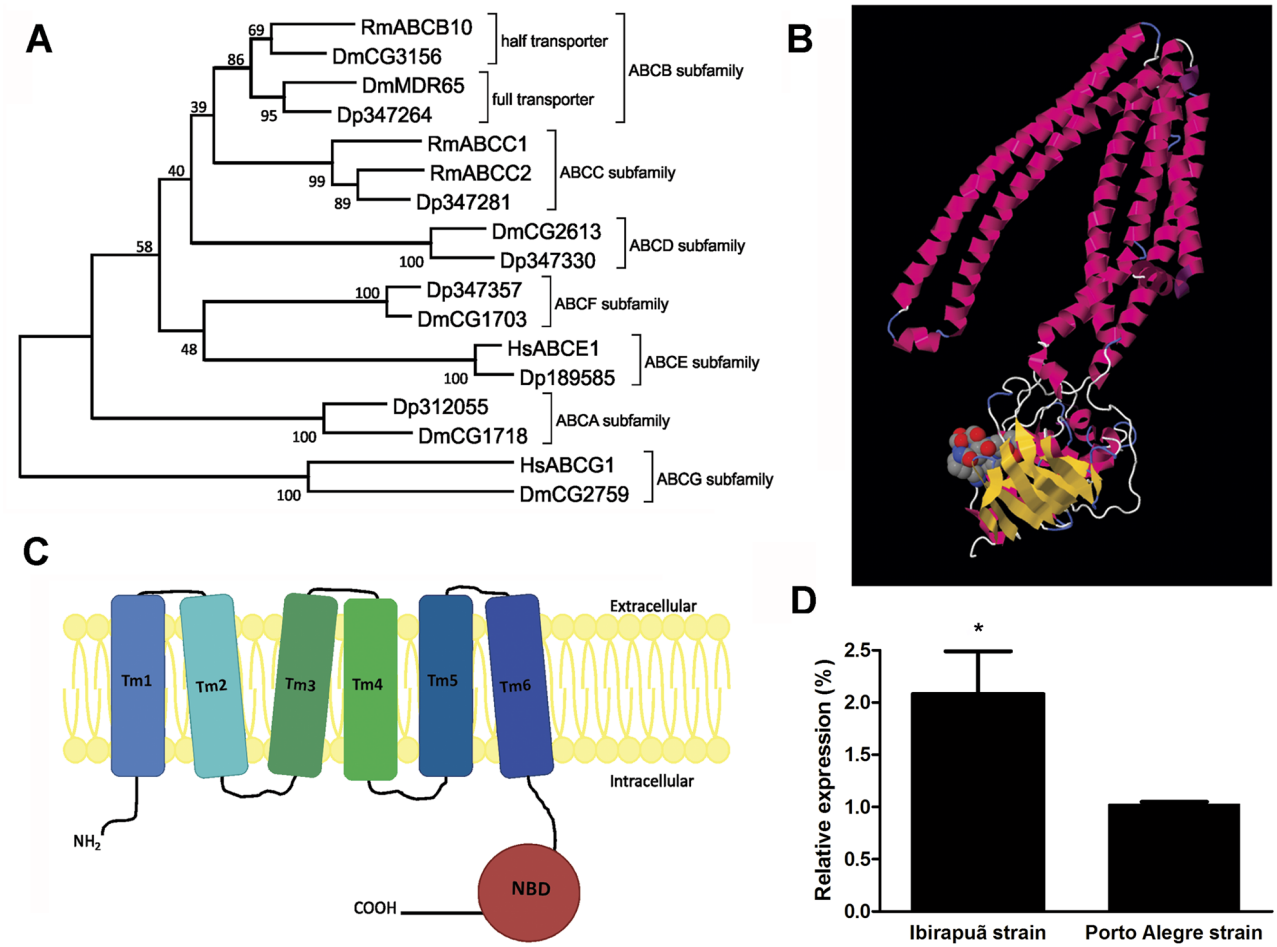
Identification and expression of ABC transporters in midgut digest cells. (A) Phylogenetic analysis based on the alignment of RmABCB10 (bold) with ABC transporters from other organisms. Dm: *D*. *melanogaster*, Dp: *D*. *pulex*, Hs: *H*. *sapiens*, Rm: *R*. *microplus*. Accession numbers of the sequences are described in Methods. (B) Proposed scheme showing the secondary structure of the RmABCB10 protein consisting of six transmembrane domains (Tm1 to Tm6) and one cytosolic nucleotide-binding domain (NBD). (C) Predicted tertiary structure based on the amino acid sequence of the RmABCB10 protein based on the known structure of human ATP-binding cassette sub-family B member 10 (2YL4). (D) The relative expression on RmABCB10 was evaluated by qPCR in digest cells from both sensitive and resistant strains dissected 3 days ABM. The mean ± SEM are shown (n = 3); (**) represents p < 0.05 (one-way ANOVA followed by Tukey’s test).

**Fig 7:**
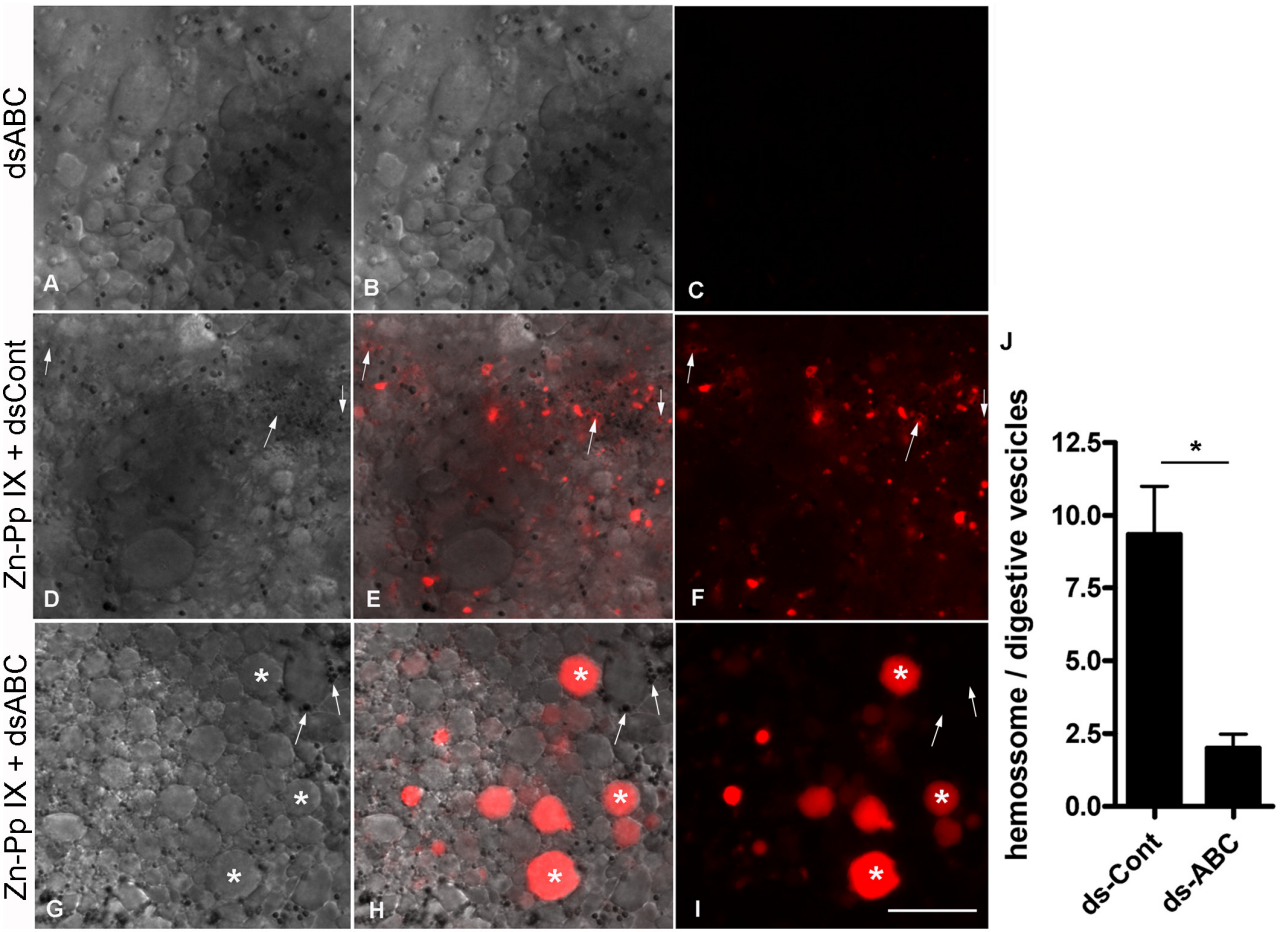
ABC transporter silencing impairs Zn-Pp IX traffic in digest cells. Partially engorged females were collected from cattle and were artificially fed with blood supplemented with dsABC (A-C), with Zn-Pp IX plus dsCont (D-F) or with Zn-Pp IX plus dsABC (G-I). In all cases, the blood meal contained 0.5% DMSO (v/v). After 72 h ABM, digest cells were detached from the tissue, and differential interference contrast (DIC) (A, D and G) and Zn-Pp IX fluorescence images (C, F and I) were acquired. Merged images are shown in B, E and H. The white arrows indicate hemosomes (small vesicles) exhibiting a Zn-Pp IX signal in panels A_F; in panels G-I, some hemosomes are indicated, but with no fluorescence associated; white asterisks show digestive vesicles within the Zn-Pp IX signal. The scale bars are 20 μm in all images. The ratio of the number of Zn-PP positive hemosomes to digestive vesicles was measured in 15 randomly chosen images from each condition, obtained from three independent experiments (J). Data shown are mean ± SEM; * means p value < 0.002 (Student’s *t* test).

Because the RmABCB10 transporter has been implicated in ivermectin resistance [25], we analyzed its involvement in amitraz resistance and heme trafficking in digest cells. Based on the partial sequence available, the full-length sequence of RmABCB10 was obtained with rapid amplification of cDNA ends (RACE-PCR). A functional ABC transporter requires two transmembrane domains with six membrane-spanning α-helices (TMD) each and two nucleotide-binding domains (NBD) units. This functional unit may be present within a single polypeptide chain (‘full transporters’), or may require the formation of a membrane-bound homo- or heterodimer of ‘half transporters’ [47]. A phylogenetic analysis comparing RmABCB10 with transporters from different ABC subfamilies places this transcript in the half-transporter group of the B subfamily (Fig 6A). An analysis of the deduced amino acid sequence found a C-terminal nucleotide-binding domain (NBD) with typical Walker A, Walker B and ABC signature motifs (Fig 6B and S1 Fig) and a N-terminal transmembrane domain (TMD) (Fig 6B and S1 Fig) with six membrane-spanning α-helices (Fig 6C). An analysis of the transcription levels in digest cells by real-time PCR showed significantly higher expression of RmABCB10 in the amitraz-resistant strain Ibirapuã than in the sensitive strain POA (Fig 6D). To further test the involvement of RmABCB10, the main up-regulated ABC transporter in amitraz resistant tick strains [25], in heme trafficking, partially engorged females were artificially fed with blood enriched with RmABCB10 dsRNA, which resulted in efficient down-regulation of RmABCB10 mRNA in the midgut (S2 Fig). When Zn-Pp IX was added to the blood meal together with the RmABCB10 dsRNA, a reduction in the labeling of hemosomes was observed in parallel with an increase in the labeling of digestive vacuoles (Fig 7). These data, together with the higher capacity of the Ibirapuã-strain hemosomes to sequester Sn-Pp IX and amitraz, strongly support the involvement of the same ABC transporter system in the detoxification of both heme and pesticides.

## Discussion

**Fig 8:**
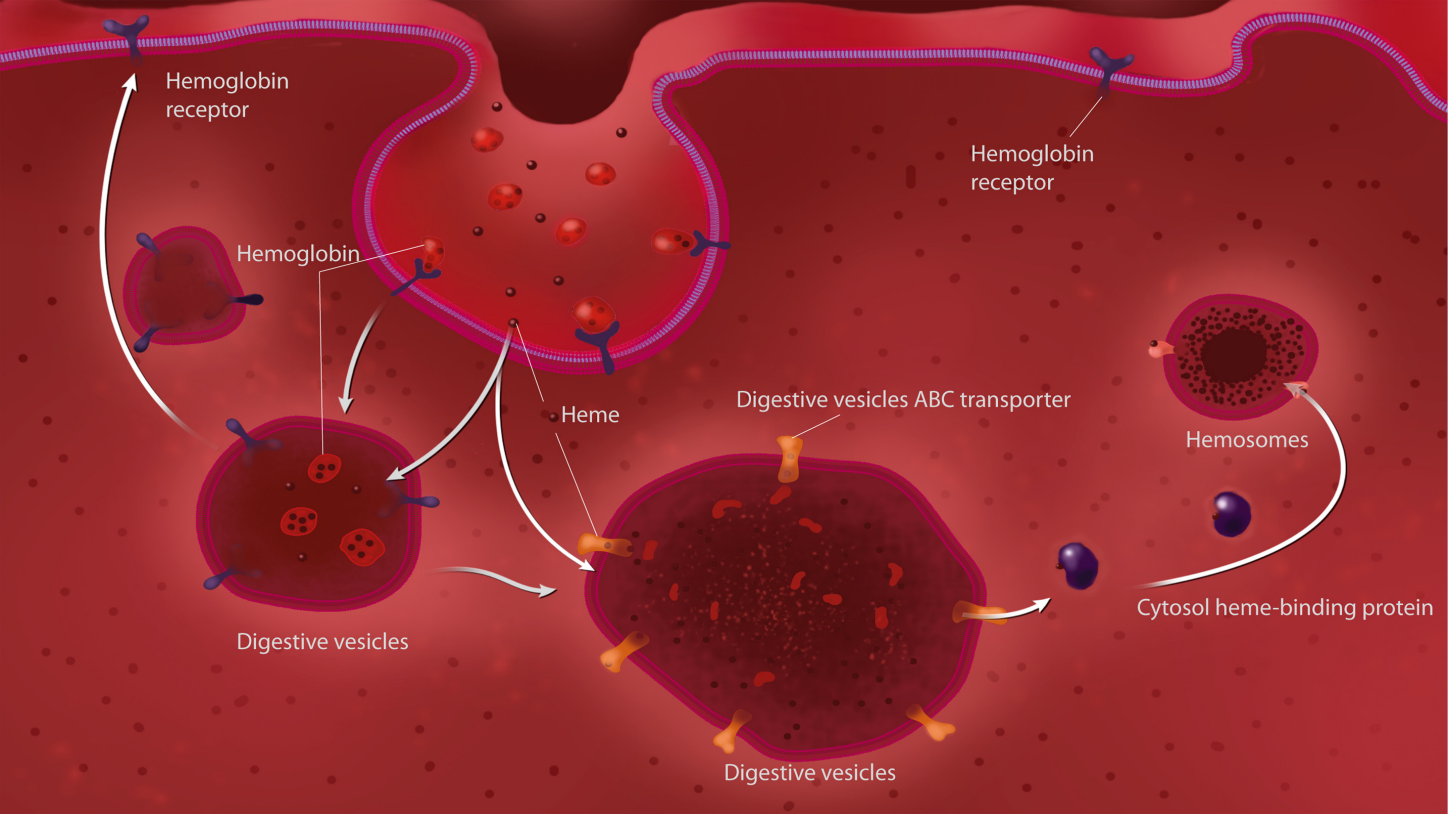
Heme traffic pathway in the digest cell of the cattle tick *Rhipicephalus microplus*. This proposed model integrates data reported here on the role of ABC transporters and results from previous reports describing the uptake of hemoglobin, followed by export of heme from the digestive vesicle to the cytosol and formation of heme aggregates in the hemosome [6, 28].

Despite the importance of heme as the prosthetic group of several fundamental enzymes and its presence in almost all organisms, the intracellular trafficking of heme is still poorly understood. ABC transporters are a family of large membrane proteins that are involved in the ATP-powered transport of a wide array of biologically relevant molecules, frequently against a concentration gradient [48]. ABC transporters have been shown to perform transmembrane heme transport in bacteria [49] and trypanosomatids [50]. In higher eukaryotes, however, other classes of transporters (non-ABC) have been implicated in heme transport across cellular membranes [10, 12, 13]. The participation of an ABC transporter in heme metabolism in typical eukaryotic cells has been shown in mitochondria, where an ABC transporter was implicated in the uptake of a heme precursor, coproporphyrinogen III, but not in heme transport directly [51]. ABCG2, also known as Breast Cancer Resistance Protein (BCRP) was shown to bind heme and to export a heme-analog, Zn-MesoPorphyrin [15]. However, it has been questioned if this protein works under physiological conditions as a heme transporter, as it can transport a wide range of substrates [52]. Recently, an ABC transporter (an ABCC/mrp) protein was shown to work as a heme exporter, which silencing resulted in an embryonic lethal phenotype in *C*. *elegans* and compromised erythropoiesis in zebrafish [16]. In the midgut of tick digest cells, we previously identified specialized organelles called hemosomes that are dedicated to the accumulation of large amounts of heme released during the digestion of a blood meal [28]. Hemosome formation is accomplished by a pathway that starts with specific endocytosis of hemoglobin [28], followed by the removal of heme from the hemoglobin polypeptide chain inside an acidic digestive vacuole, the subsequent transfer of heme to the cytosol, and finally, the uptake of heme by hemosomes [6]. Heme can account for up to 90% of the dry weight composition of hemosomes [28]. Here, we show evidence that an ABC transporter, the RmABCB10, is involved in heme transport in the digestive vesicles membranes, and is part of a trafficking pathway that leads to heme sequestration into hemosomes, which major features are summarized in a schematic model in Fig 8. The presence of an ABC transporter in the digestive vesicle was demonstrated by immunoreactivity with specific antibody against PgP-type ABC ATPase, together with the identification of ATPase enzymatic activity by cytochemistry and the accumulation of Rhodamine 123, both of which were inhibited by CsA (Figs 1–3). Direct demonstration of ABC-dependent SnPp-IX transport by fluorescence microscopy and HPLC analysis provides evidence that this mechanism is involved in the accumulation of heme (Figs 4 and 5). There are a number of reports relating ABC transporters to heme metabolism, showing that cells lacking BCRP/ABCG2 or ABCB6 accumulate porphyrin [51, 53]. ABCB6 is located on the mitochondrial outer membrane [54], whereas the BCRP protein is localized to inner mitochondrial cristae [55]; both are thought to participate in the uptake of heme precursors into the mitochondria, fueling the final steps of the heme biosynthesis pathway. As already mentioned above, a role of ABCG2 in heme transport has also been hypothesized [15, 52, 56]. In the present work, we observed inhibition of transport of metalloporphyrin to the hemosomes after RmABCB10 dsRNA silencing, together with accumulation in the digestive vesicles (Fig 7), a result that strongly suggests that this enzyme is located in the membrane of the digestive vesicles. In addition to this transporter, the heme traffic pathway in the digestive outlined previously postulates the existence of one transporter at the hemosome membrane, and data obtained here are compatible with the presence of RmABCB10 also in the hemosome membrane, as the cytochemical location of the enzyme showed the presence of ATPase activity in the hemosome (Fig 3). However, we cannot exclude the possibility that other ABC transporters, distinct from RmABCB10 –or even transporters belonging to other protein family—are also involved in the transport of heme along the digest cell pathway. Moreover, the effect of CsA (Fig 4) and RmABCB10 silencing (Fig 7) on metalloporphyrin traffic are not identical, as CsA blocks also accumulation in the digestive vesicle. This result suggest that another ABC transporter (distinct from RmABCB10) is needed to allow heme accumulation into the digestive vesicle. These findings point to a scenario that resembles what has been shown for heme transport in the gut of *C*.*elegans*, where heme is transferred into a vesicle in the endosome-lysosomal pathway, with one transporter needed to take heme to this compartment (HRG-4) and another (HRG-1) to direct it to the cytosol [13, 57, 58]. However, in spite of the overall similarity in the general features of the pathway, a major difference needs attention that is the fact that the transporters involved in each organism belong to distinct protein families. This might be attributed to the exceptionally high amount of heme produced by a blood meal, which possibly demanded a dedicated group of proteins as well as the unique digestive system of ticks that is based on intracellular digestion of food instead of the most common use of an extracellular pool of hydrolases in the gut luminal cavity. Also is worth to be mentioned that ticks are probably the oldest hematophagous animals, possibly originated in the middle Permian (260–270 MYA) [59], but that, accordingly to some authors, may date as early as the Devonian (417–362 MYA), a time interval that would be enough to allow the development of such a complex heme traffic pathway. In the last few years, a set of proteins conserved among metazoans have been implicated in heme intracellular traffic, specially by the work of Hamza’s group [60]. The hypothesis proposed here that ticks have acquired a specialized heme transport machinery brings about another question that is what is the role of those “pan-metazoa” heme transport proteins in ticks.

As already mentioned, the pathway that goes to a hemoglobin-degrading digestive vesicle to the hemosome is dedicated to heme detoxification in this obligate blood-feeding arthropod. The cattle tick has been shown to lack heme biosynthesis [61], which excludes any connection of the heme cellular trafficking route studied here to this metabolic pathway. Here, we show that heme transportation to the cytosol of tick digest cells is performed by an ABC enzyme. These results also indicate that this is an ancestral mechanism capable of mediating heme transport across membranes, similar to its role in bacteria [62] and trypanosomatids [50, 63] and recently extended to other eukaryotic cells [16]. However, it worth mentioning that the ABC transporter described here belongs to an ABC subfamily distinct from the ABCC5/MRP5 identified in other metazoa [16], reinforcing the hypothesis that a complete heme transport system has evolved in ticks.

ABC transporters have been primarily related to the export of toxic molecules but have also been shown to play a role in the import of nutrients and the transport of many other physiological substrates. As a consequence, a relatively large number of genes from this family are found in most eukaryotic organisms, such as in humans, where 46 genes have been identified [64], or in the mosquito genome, which contains 64 ABC genes [65]. However, as the variety of molecules that are ABC substrates is very large, these transporters frequently have a broad specificity; as a result, there are several different molecules that can be translocated by the same protein. The ABC-mediated accumulation of the acaricide amitraz inside the hemosome reported here may be due to this non-stringent specificity. Alternatively, these results could be explained by the participation of more than one ABC transporter in delivering amitraz to the hemosome, but the presence of increased heme transport and RmABCB10 expression in a tick strain resistant to acaricide [25] and in tick cell line exposed to acaricides [24] suggest that at least this transporter is used to detoxify both compounds.

As mentioned above, the most intensively studied role of ABC transporters is the detoxification of xenobiotics, as well as the capacity of ABCs to detoxify pesticides, has already been described in mammalian cells [66–68]. With respect to the detoxification of pesticides by insects and ticks, however, the involvement of ABCs has been overlooked in the past, but in the last years several reports have indicated the association of insecticide resistance to ABC transporters [21, 23]. Susceptibility of *Culex pipiens* to insecticides was increased by verapamil, thereby suggesting the participation of ABC transporters in the resistance to this insecticide [69] and more recently, transcriptome data of pyrethroid resistant *Aedes aegypti* populations revealed increased expression of ABC genes in the resistant strains [21]. Here, we clearly show ABC-dependent accumulation of amitraz in the hemosome, an intracellular organelle primarily dedicated to sequestration of heme, which is also a toxic molecule. The validity of this trafficking pathway as a potentially new mechanism of resistance stems from the fact that a tick strain resistant to this acaricide demonstrated increased transport of both amitraz and heme to the hemosome. The increased expression of the mRNA of at least one ABC in digest cells provides additional support for this hypothesis. The ABC activity observed here was associated with the membrane of the digestive vesicles. This unique location may be used to create new tick control strategies, as this ABC transporter is the first described that pumps toxic compounds into an intracellular route, leading to accumulation inside an organelle cell, and not into the external medium, such as the other ABC transporters described thus far. We believe that this particularity is due to presence in the hemosomes in the cytosol of the digestive cells, an organelle primarily dedicated to heme detoxification previously described by our group [28]. Based on this data, control strategies that can disrupt the accumulation of heme into the hemosome could also increase the sensitivity of these animals to commonly used pesticides.

Taken together, our data show that, similar to bacteria and trypanosomatid protozoa, complex eukaryotes also employ ABC transporters in the intracellular trafficking of heme and that the same mechanism can also be used in the detoxification of xenobiotics such as pesticides. These findings have potential implications in both the understanding of the drug detoxification metabolism in arthropod pests and the basic knowledge of heme trafficking within cells in eukaryotic organisms.

## Supporting Information

S1 Fig Multiple sequence alignment of the predicted amino acid sequence of the *R*. *microplus* ABC transporter RmABCB10 (AEI91123.2) and amino acid sequences of *H*. *sapiens* ABCB10 (NP036221.2), ABCB8 (NP009119.2) and ABCB7 (NP004290.2); *M*. *musculus* ABC-me (NP062425.1); *D*. *melanogaster* G3156 (NP569844.2); *D*. *pulex* 347276 (EFX65703.1); and *S*. *cerevisiae* Mdl1p (NP13289.1), Mdl2p (NP015053.2) and Atm1p (NP014030.1). Conserved motifs of the nucleotide-binding domain (NBD) are indicated by boxes, and transmembrane regions are highlighted. Alignments were performed using the MUSCLE algorithm with the default settings in MEGA 5 software.
(PDF)

S2 Fig Analysis of RNAi-mediated RmABCB10 silencing in the midgut of ticks after repletion. The relative expression of RmABCB10 was determined by quantitative PCR of the total RNA extracted from the midguts of female ticks collected 24 hours and 48 hours after repletion and injected with RmABCB10 dsRNA or control dsRNA. The points represent the percent of RmABCB10 silencing of six females from each group, and the means are indicated with a bar. Asterisks (*) denote a significant difference as determined by a one-way ANOVA followed by Tukey’s test (p≤0.05).
(PDF)
