# Supplementary material for: ATP Binding Cassette Transporter Mediates Both Heme and Pesticide Detoxification in Tick Midgut Cells
Source: PLoS One. 2015 Aug 10;10(8):e0134779. doi: 10.1371/journal.pone.0134779 (PMC4530934; doi:10.1371/journal.pone.0134779)
Supplement: S1 Fig — Conserved motifs of the nucleotide-binding domain (NBD) are indicated by boxes, and transmembrane regions are highlighted. Alignments were performed using the MUSCLE algorithm with the default settings in MEGA 5 software. (PDF) [file pone.0134779.s001.pdf]

```
                    10        20        30        40        50        60        70        80        90
           ....|....|....|....|....|....|....|....|....|....|....|....|....|....|....|....|....|....|
RmABCB10   MHAFVLSAGSQLFRQVKN-----------RKHVL-----------LRFISSKNGGV-----LSNSSAIRRVCVPPVK-------------
Dp347276   ------MALVSRLFTSPS-----------YSRQV-----------ILLVRARCSSL-------ASRSLQPASPGKSNG------------
DmCG3156   ----MLLNCTRLTHLCTT--------LRVRKSSQ-----------IRLSTSYNHHLGRCLHHRTARSLQHSLPPPNRYHSHLHPTLRMPW
Mm_ABC-me  MRAPSARALLLIPRRGPA--------VRAWAPAV-------------SSRIWLASE---W-TPLVRAWTSLIHKPGSGLRFPAPLSGLPG
HsABCB10   MRGPPAWPLRLLEPPSPAEPGRLLPVACVWAAASRVPGSLSPFTGLRPARLWGAGPALLWGVGAARRWRSGCRGGGPGASR--GVLGLAR
HsABCB8    -------MLVHLFRVGIR-------GGPFPGRLL-----------PPLRFQTFSAVRYSDGYRSSSLLRAVAHLRSQLWAHLPRAPLAPR
HsABCB7    ------MALLAMHSWR-------------WAAAA-----------AAFEKRRHSAI----------LIRPLVSVSGSGPQWRPHQLGALG
ScMdl1p    -------MIVRMIRLC-------------KGPKL-----------LRSQFASASAL-----YSTKSLFKPPMYQKAEINLIIPH------
ScMdl2p    ----MLNGRLPLLRLGIC-------RNMLSRPRL-----------AKLPSIRFRSLVTPSSSQLIPLSRLCLRSPAVGKSLILQSF----
ScAtm1p    ---------MLLLPRCPV---------------I-----------GRIVRSKFRS-------GLIRNHSPVIFTVSKLSTQRPLLFNS--

           ....|....|....|....|....|....|....|....|....|....|....|....|....|....|....|....|....|....|
RmABCB10   -----------------------SLASVVLCR---------------------------ACSNARRVDSSELRR-LLRQARPE-----RF
Dp347276   ----------GA--NCSWRFFSSTSALLRNSGLKTA-----------------------EKVKPKLKS-EEVRR-LIGLAKPE-----KY
DmCG3156   -----WCMRRGFAQSSKWLARNAKANLPTAVGAGIVPA-------------------------PVKMGRSQYGR-LLSLTKSE-----KW
Mm_ABC-me  -GVGQWATSSGA--RRCWVLAGPRAAHPLFARLQGAAATG------------VRDLGNDSQRRPAATGRSEVWK-LLGLVRPE-----RG
HsABCB10   -LLGLWARGPGS--CRCGAFAGPGAPRLPRARFPGGPAAAAWAGDEAWRRGPAAPPGDKGRLRPAAAGLPEARK-LLGLAYPE-----RR
HsABCB8    WSPSAWCWVGGALLGPMVLSKHPHLCLVALCEAEEAPP---------------------ASSTPHVVGSRFNWKLFWQFLHPH-----LL
HsABCB7    -TARAYQQIPESLKSITWQRLGKGNSGQFLDAAKALQVWPLIEKRTCWHGHAGGGLHTDPKEGLKDVDTRKIIKAMLSYVWPKDRPDLRA
ScMdl1p    --------------RKHFLLRSIRLQSDIAQGKKSTKPTLKL-----------------SNANSKSSGFKDIKR-LFVLSKPE-----SK
ScMdl2p    --------------RCNSSKTVPETSLPSASPISKGSARSAH-----------------AKEQSKTDDYKDIIR-LFMLAKRD-----WK
ScAtm1p    -AVNLWNQAQKDITHKKSVEQFSSAPKVKTQVKKTSKA-------------------------PTLSELKILKD-LFRYIWPKGNNKVRI

           ....|....|....|....|....|....|....|....|....|....|....|....|....|....|....|....|....|....|
RmABCB10   RLGAAVLLLFVSSSVMIAFPFCIGKVIDVI--YTSSDNAELR-AN-------LNWICKLLTGVVVIGGLANFGRVY-------LMNSSAQ
Dp347276   RLLGGIGLLFVSSAITMVIPFAIGKVIDII-----SANQESMVEN-------LTNVSLILVGIFIFGAACNFGRTY-------LMSVAGE
DmCG3156   VLTAGIGCLVVSSAITMSVPLFLGKVIDVV--FNKSGMDSAAMAK-------LGEYSVLLFGIFVLGGFANFARVH-------LFGNAAL
Mm_ABC-me  RLSAAVGFLAVSSVITMSAPFFLGRIIDVI--YTNPSEGYG--DS-------LTRLCAVLTCVFLCGAAANGIRVY-------LMQSSGQ
HsABCB10   RLAAAVGFLTMSSVISMSAPFFLGKIIDVI--YTNPTVDYS--DN-------LTRLCLGLSAVFLCGAAANAIRVY-------LMQTSGQ
HsABCB8    VLGVAVVLALGAALVNVQIPLLLGQLVEVVAKYTRDHVGSFMTES--------QNLSTHLLILYGVQGLLTFGYLV-------LLSHVGE
HsABCB7    RVAISLGFLGGAKAMNIVVPFMFKYAVDSL--NQMSGNMLNLSDA-------PNTVATMATAVLIGYGVSRAGAAFFNEVRNAVFGKVAQ
ScMdl1p    YIGLALLLILISSSVSMAVPSVIGKLLDLA--SESDGEDEEGSKSNKLYGFTKKQFFTALGAVFIIGAVANASRII-------ILKVTGE
ScMdl2p    LLLTAILLLTISCSIGMSIPKVIGIVLDTL--KTSSGSDFFDLKI-PIFSLPLYEFLSFFTVALLIGCAANFGRFI-------LLRILSE
ScAtm1p    RVLIALGLLISAKILNVQVPFFFKQTIDSM--------NIAWDDP-------TVALPAAIGLTILCYGVARFGSVLFGELRNAVFAKVAQ
                       Tm1                                                      Tm2
           ....|....|....|....|....|....|....|....|....|....|....|....|....|....|....|....|....|....|
RmABCB10   RIINSLRKQAHASLMQQEVAFFDRNRTGDLITRLSSDTALVGMSLTQNISDGLRFAVAVFGGVGMMLY-TSPQLSLVGLSVVPPVAIISF
Dp347276   RITQTMRRNVYAAIVKQDVKFFDKNQTGELVNRLSTDTTLVSKCVTANLADGIRSSALVVSAISMMFY-TSPQIALVGLSIVPPVAGLTI
DmCG3156   RIVRSLRSRLYRSMLMQEVGWFDTKGTGELINRLSNDTLMVGTSLSQNVSDGLRSVAMIGVGTGMMIY-TSPQLAAVSALVVPAMAGMAI
Mm_ABC-me  SIVNRLRTSLFSSILRQEVAFFDKTRTGELINRLSSDTALLGRSVTENLSDGLRAGAQASVGVGMMFF-VSPSLATFVLSVVPPISVLAV
HsABCB10   RIVNRLRTSLFSSILRQEVAFFDKTRTGELINRLSSDTALLGRSVTENLSDGLRAGAQASVGISMMFF-VSPNLATFVLSVVPPVSIIAV
HsABCB8    RMAVDMRRALFSSLLRQDITFFDANKTGQLVSRLTTDVQEFKSSFKLVISQGLRSCTQVAGCLVSLSM-LSTRLTLLLMVATPALMGVGT
HsABCB7    NSIRRIAKNVFLHLHNLDLGFHLSRQTGALSKAIDRGTRGISFVLSALVFNLLPIMFEVMLVSGVLYYKCGAQFALVTLGTLGTYTAFTV
ScMdl1p    RLVARLRTRTMKAALDQDATFLDTNRVGDLISRLSSDASIVAKSVTQNVSDGTRAIIQGFVGFGMMSF-LSWKLTCVMMILAPPLGAMAL
ScMdl2p    RVVARLRANVIKKTLHQDAEFFDNHKVGDLISRLGSDAYVVSRSMTQKVSDGVKALICGVVGVGMMCS-LSPQLSILLLFFTPPVLFSAS
ScAtm1p    NAIRTVSLQTFQHLMKLDLGWHLSRQTGGLTRAMDRGTKGISQVLTAMVFHIIPISFEISVVCGILTYQFGASFAAITFSTMLLYSIFTI
                                                                   Tm3                    Tm4
           ....|....|....|....|....|....|....|....|....|....|....|....|....|....|....|....|....|....|
RmABCB10   AFARRLRQVAADVQTRLAESSATAEEQLSNIRTVRAFTKEKFEMNRYSVGLTRLLDKVNTETRLRAVFFGCTGATGNMIVLAVLYYGGIL
Dp347276   VYGRFVKKITKKVQDALAQATQVTEERISNIRTVRAFGQEKREFERYNEQIDHVLTLGYKEAKARGTFFALTGFAGNAIIISGLYYGGLM
DmCG3156   VYGRYVRRITKVELDKYAEIMKFAEERFGNVKTVKTFCREQQEVAAFDGKLDEALQIGYKETRARAIFFGLTGFCGNFIIISVLYYGGTL
Mm_ABC-me  IYGRYLRKLSKATQDSLAEATQLAEERIGNIRTIRAFGKEMTEVEKYTGRVDQLLQLAQKEALARAGFFGAAGLSGNLIVLSVLYKGGLL
HsABCB10   IYGRYLRKLTKVTQDSLAQATQLAEERIGNVRTVRAFGKEMTEIEKYASKVDHVMQLARKEAFARAGFFGATGLSGNLIVLSVLYKGGLL
HsABCB8    LMGSGLRKLSRQCQEQIARAMGVADEALGNVRTVRAFAMEQREEERYGAELEACRCRAEELGRGIALFQGLSNIAFNCMVLGTLFIGGSL
HsABCB7    AVTRWRTRFRIEMNKADNDAGNAAIDSLLNYETVKYFNNERYEAQRYDGFLKTYETASLKSTSTLAMLNFGQSAIFSVGLTAIMVLASQG
ScMdl1p    IYGRKIRNLSRQLQTSVGGLTKVAEEQLNATRTIQAYGGEKNEVRRYAKEVRNVFHIGLKEAVTSGLFFGSTGLVGNTAMLSLLLVGTSM
ScMdl2p    VFGKQIRNTSKDLQEATGQLTRVAEEQLSGIKTVQSFVAEGNELSRYNVAIRDIFQVGKTAAFTNAKFFTTTSLLGDLSFLTVLAYGSYL
ScAtm1p    KTTAWRTHFRRDANKADNKAASVALDSLINFEAVKYFNNEKYLADKYNGSLMNYRDSQIKVSQSLAFLNSGQNLIFTTALTAMMYMGCTG
                                                                                Tm5
           ....|....|....|....|....|....|....|....|....|....|....|....|....|....|....|....|....|....|
RmABCB10   MSDGRLTVGSLSSFLLYAAYVGVTIGGLGGFFTEATKALGASKKVWEIADRVPTLPNFG-G--LALSNLQGQVEFHNVTFAYSSRPEIEV
Dp347276   VADSSLTVGQLSAFLLYAAYVGISMGGLSSSYSEMMKGLGASTRIWQLIDTRPDIPISG-GPVIPAGQFEGSIRFRDLSFAYPTRSDVPI
DmCG3156   VLQDSLTIGALTAFMLYAGYVAISMNGLSNFYSQLNKGIGASERIWEILDRECSIPIDK-G-VVPLEKPVGEVGFQNVFFTFPTRPESAV
Mm_ABC-me  MGSAHMTVGELSSFLMYAFWVGLSIGGLSSFYSELMKGLGAGGRLWELLERQPRLPFNE-GMVLDEKTFQGALEFRNVHFTYPARPEVSV
HsABCB10   MGSAHMTVGELSSFLMYAFWVGISIGGLSSFYSELMKGLGAGGRLWELLEREPKLPFNE-GVILNEKSFQGALEFKNVHFAYPARPEVPI
HsABCB8    VAGQQLTGGDLMSFLVASQTVQRSMANLSVLFGQVVRGLSAGARVFEYMALNPCIPLSG-GCCVPKEQLRGSVTFQNVCFSYPCRPGFEV
HsABCB7    IVAGTLTVGDLVMVNGLLFQLSLPLNFLGTVYRETRQALIDMNTLFTLLKVDTQIKDKVMASPLQITPQTATVAFDNVHFEY--IEGQKV
ScMdl1p    IQSGSMTVGELSSFMMYAVYTGSSLFGLSSFYSELMKGAGAAARVFELNDRKPLIRPTI-G-KDPVSLAQKPIVFKNVSFTYPTRPKHQI
ScMdl2p    VLQSQLSIGDLTAFMLYTEYTGNAVFGLSTFYSEIMQGAGAASRLFELTDRKPSISPTV-G--HKYKPDRGVIEFKDVSFSYPTRPSVQI
ScAtm1p    VIGGNLTVGDLVLINQLVFQLSVPLNFLGSVYRDLKQSLIDMETLFKLRKNEVKIKNAE-RPLMLPENVPYDITFENVTFGY--HPDRKI
                  Tm6
```

```
            ....|....|....|....|....|....|....|....|....|....|....|....|....|....|....|....|....|....|
RmABCB10    LKKLNLSVPAGSVLAIVGPSGQGKSTLASLLLRLYDPTSGTVTLDGIDIRELDPHFLRMH-VGIVSQEPTLFATSIFENILYGAKNMEES
Dp347276    FNNLNLSIPAGSVTAIVGSSGSGKSTIAALLLRFYDPTSGQVLVDNHSIKDLDPQWLRAN-IGTVSQEPSLFSCSIRENILYGAIDPNSV
DmCG3156    LTDFSLNLMPGTTTAVVGRSGSGKTTIALLMLRLYDPQGGTVHLDGIDLRTVNPQWLRNN-IGAVSQEPVLFSCSIRENILYGANPGETP
Mm_ABC-me   FQDFSLSIPSGSVTALVGPSGSGKSTVVSLLLRLYDPNSGTVSLDGHDIRQLNPVWLRSK-IGTVSQEPVLFSCSVAENIAYGADNLSSV
HsABCB10    FQDFSLSIPSGSVTALVGPSGSGKSTVLSLLLRLYDPASGTISLDGHDIRQLNPVWLRSK-IGTVSQEPILFSCSIAENIAYGADDPSSV
HsABCB8     LKDFTLTLPPGKIVALVGQSGGGKTTVASLLERFYDPTAGVVMLDGRDLRTLDPSWLRGQVVGFISQEPVLFGTTIMENIRFG---KLEA
HsABCB7     LSGISFEVPAGKKVAIVGGSGSGKSTIVRLLFRFYEPQKGSIYLAGQNIQDVSLESLRRA-VGVVPQDAVLFHNTIYYNLLYG---NISA
ScMdl1p     FKDLNITIKPGEHVCAVGPSGSGKSTIASLLLRYYDVNSGSIEFGDEDIRNFNLRKYRRL-IGYVQQEPLLFNGTILDNILYCIPPEIAE
ScMdl2p     FKNLNFKIAPGSSVCIVGPSGRGKSTIALLLLRYYNPTTGTITIDNQDISKLNCKSLRRH-IGIVQQEPVLMSGTIRDNITYGL--TYTP
ScAtm1p     LKNASFTIPAGWKTAIVGSSGSGKSTILKLVFRFYDPESGRILINGRDIKEYDIDALRKV-IGVVPQDTPLFNDTIWENVKFG---RIDA
                         Walker A
            ....|....|....|....|....|....|....|....|....|....|....|....|....|....|....|....|....|....|
RmABCB10    SKEDVIRAASEANALEFIEDLPDGFNTMVGERGILLSGGQKQRIAIARAILRDPQVLILDEATSSLDAVSERAVQEALKKLMV--GRTVL
Dp347276    SEEQVIQAAKEANAWSFIQKCPQGLDTVVGERGVLLSGGQRQRIAIARAIVKNPRILLLDEATSALDAESESLIQEALERVTK--GRTVL
DmCG3156    SPERLQQVIEDANVSQFTDQLPDGLDTLVGQRGMMLSGGQKQRVAIARALIKNPAILILDEATSALDAVSENLVQNALDNLIQ--GRTVL
Mm_ABC-me   TAQQVERAAEVANAAEFIRSFPQGFDTVVGEKGILLSGGQKQRIAIARALLKNPKILLLDEATSALDAENEHLVQEALDRLME--GRTVL
HsABCB10    TAEEIQRVAEVANAVAFIRNFPQGFNTVVGEKGVLLSGGQKQRIAIARALLKNPKILLLDEATSALDAENEYLVQEALDRLMD--GRTVL
HsABCB8     SDEEVYTAAREANAHEFITSFPEGYNTVVGERGTTLSGGQKQRLAIARALIKQPTVLILDEATSALDAESERVVQEALDRASA--GRTVL
HsABCB7     SPEEVYAVAKLAGLHDAILRMPHGYDTQVGERGLKLSGGEKQRVAIARAILKDPPVILYDEATSSLDSITEETILGAMKDVVK--HRTSI
ScMdl1p     QDDRIRRAIGKANCTKFLANFPDGLQTMVGARGAQLSGGQKQRIALARAFLLDPAVLILDEATSALDSQSEEIVAKNLQRRVE-RGFTTI
ScMdl2p     TKEEIRSVAKQCFCHNFITKFPNTYDTVIGPHGTLLSGGQKQRIAIARALIKKPTILILDEATSALDVESEGAINYTFGQLMKSKSMTIV
ScAtm1p     TDEEVITVVEKAQLAPLIKKLPQGFDTIVGERGLMISGGEKQRLAIARVLLKNARIMFFDEATSALDTHTEQALLRTIRDNFTSGSRTSV
                                                Signature      Walker B
            ....|....|....|....|....|....|....|....|....|....|....|....|....|....|....|....|....|....|
RmABCB10    TIAHRLSTIRRADKIAVL-KAGTVVEHGTYEQLMGIADGLFRRLVEHQLHDERMSG----------------------------------
Dp347276    TIAHRLSTIRKASQIAVL-EHGQVVELGTYEALVSIPDGVFKKLVELQTIGLAAA-----------------------------------
DmCG3156    TIAHRLSTIRNADQIAVL-SDGKIVEQGSYNELMGIQEGVFRELVASQAFGSRN------------------------------------
Mm_ABC-me   IIAHRLSTIKNANFVAVL-DHGKICEHGTHEELLLKPNGLYRKLMNKQSFLSYNGAEQFLEPARA-------------------------
HsABCB10    VIAHRLSTIKNANMVAVL-DQGKITEYGKHEELLSKPNGIYRKLMNKQSFISA-------------------------------------
HsABCB8     VIAHRLSTVRGAHCIVVM-ADGRVWEAGTHEELL-KKGGLYAELIRRQALDAPRTAAPPPKKPEGPRSHQHKS-----------------
HsABCB7     FIAHRLSTVVDADEIIVL-DQGKVAERGTHHGLLANPHSIYSEMWHTQSSRVQNHDNPKWEAKKENISKEEERKKLQEEIVNSVKGCGNC
ScMdl1p     SIAHRLSTIKHSTRVIVLGKHGSVVETGSFRDLIAIPNSELNALLAEQQDEEGKGGVIDLDNSVAREV----------------------
ScMdl2p     SIAHRLSTIRRSENVIVLGHDGSVVEMGKFKELYANPTSALSQLLNEKAAPGPSDQQLQIEKVIEKEDLNESKEHDDQKKDDNDDNDNNH
ScAtm1p     YIAHRLRTIADADKIIVL-DNGRVREEGKHLELLAMPGSLYRELWTIQEDLDHLENELKDQQEL--------------------------

            ....|....|....|....|....|....|....|....|...
RmABCB10    -------------------------------------------
Dp347276    -------------------------------------------
DmCG3156    -------------------------------------------
Mm_ABC-me   -------------------------------------------
HsABCB10    -------------------------------------------
HsABCB8     -------------------------------------------
HsABCB7     SC-----------------------------------------
ScMdl1p     -------------------------------------------
ScMdl2p     DNDSNNQSPETKDNNSDDIEKSVEHLLKDAAKEANPIKITPQP
ScAtm1p     -------------------------------------------
```
