# Supplementary figures and images for: ATP Binding Cassette Transporter Mediates Both Heme and Pesticide Detoxification in Tick Midgut Cells
Source: PLoS One. 2015 Aug 10;10(8):e0134779. doi: 10.1371/journal.pone.0134779 (PMC4530934; doi:10.1371/journal.pone.0134779)

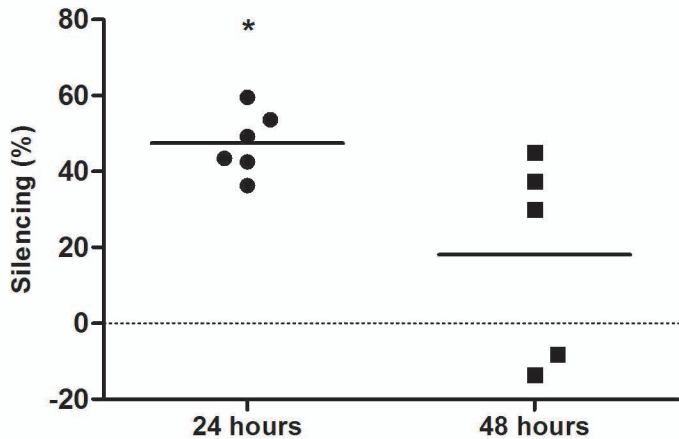

Supplement: S2 Fig — The relative expression of RmABCB10 was determined by quantitative PCR of the total RNA extracted from the midguts of female ticks collected 24 hours and 48 hours after repletion and injected with RmABCB10 dsRNA or control dsRNA. The points represent the percent of RmABCB10 silencing of six females from each group, and the means are indicated with a bar. Asterisks (*) denote a significant difference as determined by a one-way ANOVA followed by Tukey’s test (p≤0.05). (PDF) [file pone.0134779.s002.pdf]
